# Comparison of Baska Mask Versus Proseal Laryngeal Mask Airway in Elective Surgeries Under General Anaesthesia: A Randomized Clinical Trial

**DOI:** 10.7759/cureus.37366

**Published:** 2023-04-10

**Authors:** Jophy Jose, Nirmala Devi Kagalkar, Milind M Kattimani, Anusha Suntan

**Affiliations:** 1 Anesthesiology, Shri BM Patil Medical College, Hospital, and Research Centre, Bijapur Lingayat District Educational (BLDE) University (Deemed to be University), Vijayapura, IND

**Keywords:** advanced airway management, oropharyngeal seal pressure, general anesthesia, proseal laryngeal mask airway, baska mask

## Abstract

Background

Baska Mask (BM) is a third-generation supraglottic airway device with a self-inflating cuff. This study aimed to evaluate the efficacy of the BM compared to ProSeal laryngeal mask airway (PLMA) regarding insertion time, ease of insertion, and oropharyngeal seal pressure in patients undergoing elective surgeries under general anesthesia for less than two hours.

Methods

This prospective, randomized, double-blind comparative study was done on 64 patients randomly divided into two groups, with 32 patients in the PLMA group (Group A) and 32 in the BM group (Group B). Individuals with a body mass index (BMI) of more than 30, a history of nausea/vomiting, or pharyngeal pathology were excluded from the trial. After induction with propofol 3-4 mg/kg, fentanyl 1-2 mcg/kg, and the neuromuscular blockade was achieved with atracurium 0.5 mg/kg, patients were inserted with either BM (n= 32) or PLMA (n= 32). The primary outcome measure was the time taken for insertion and ease of insertion. Secondary outcome measures included the number of attempts, oropharyngeal seal pressure (OSP), and laryngopharyngeal morbidity (trauma to lips, blood staining, and sore throat) immediately and 24 hours postoperatively.

Results

Demographic data were comparable and statistically insignificant. Regarding time and ease of insertion, the BM could be inserted in a lesser time of 24±1.136 seconds compared to PLMA which took 28.59±1.682 seconds, with a high success rate in the first attempt which was statistically significant. The BM provided a higher OSP (31.34 +1.638 cmH2O) when compared to PLMA (24.81±1.469 cmH2O) and was statistically significant. Complications associated with insertion trauma to the lip, blood staining, and sore throat were more in PLMA (15.6%, 15.6 %, 9.4%, respectively) compared to the BM (6.3%, 3.1%, 3.1%, respectively), and statistically insignificant.

Conclusion

BM had higher first-attempt successful insertion with better OSP compared to PLMA in patients under controlled ventilation.

## Introduction

Supraglottic airway devices (SAD) have become a desirable substitute for tracheal intubation in patients with low risk of aspiration and conditions, who do not require high airway pressure for ventilation, as these devices can be inserted quickly and without complications associated with tracheal intubation [1]. Anesthesia changed drastically in 1988 with the entry of Archie Brain's Laryngeal Mask Airway (LMA®) Classic™ (Teleflex Incorporated, Wayne, Pennsylvania, United States) into clinical use [2]. Since then, the industry has seen an enormous increase in novel extraglottic airway devices (EAD). The term SAD describes a diverse range of medical apparatuses that can serve as a pathway for breathing, oxygenation, and the administration of anesthetic gases [3,4]. SADs are the most common modality of airway management in short surgical procedures [5].

ProSeal™ LMA (PLMA) (Teleflex Incorporated) is a sophisticated laryngeal mask device with two cuffs and an integrated bite block. A redesigned cuff enhances the laryngeal seal, enabling ventilation at significantly higher airway pressures. Its esophageal drainage tube, lateral to the main airway tube, lowers the danger of gastric insufflation and pulmonary aspiration [6]. It is a reusable device with an extra inflated dorsal cuff, integrated biting block, and a gastric drain tube as a bypass conduit for regurgitated stomach contents. Though PLMA is considered a gold standard second-generation SAD as a suitable alternative to the endotracheal tube (ETT) for airway management, it has some limitations; overinflation of its cuff can displace it and diffusion of nitrous oxide into the cuff during anaesthesia which can increase intracuff pressure and oropharyngeal morbidity [7].

The recently developed third-generation Baska Mask (BM) (Proact Medical Systems, Frenchs Forest, New South Wales, Australia), a single-use SAD, has a silicone, non-inflatable cuff. Each size, unlike PLMA, has its color code. Tissue damage may be reduced with a non-inflatable cuff. Positive pressure ventilation increases the seal and reduces leakage. The cuff's dorsal surface is molded to deflect any oropharyngeal content away from the glottis and towards side channels to which suction can be administered, reducing the risk of pulmonary aspiration. The mask contains an upper esophageal intake. Additionally, an integrated bite block and an extended hand tab attached to the cuff permit the operator to adjust the degree of bending of the devices during insertion [6].

The relative efficacy of SAD in terms of ease of application, adequacy of the ceiling, and post-insertion complications remain unclear. Hence, in this study, we were comparing PLMA and BM. The primary objective of this study was to compare the time taken for insertion and ease of insertion to determine the first-pass success rate. Secondary objectives were oropharyngeal seal pressure (OSP), and complications such as trauma to the lip, sore throat, and dysphagia between the two groups.

## Materials and methods

**Figure 1:**
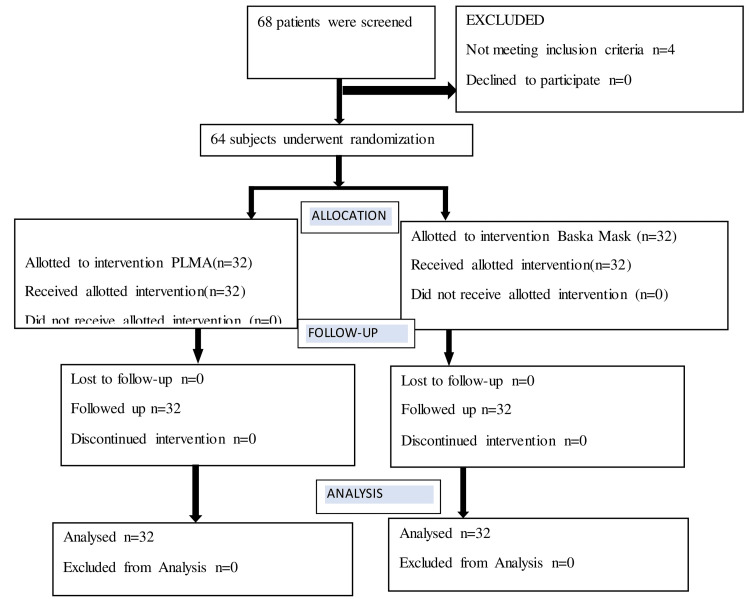
CONSORT Diagram CONSORT: Consolidated Standard of Reporting Trials; n: number of patients; PLMA: ProSeal Laryngeal Mask Airway

The study was conducted at the Department of Anesthesiology, Shri BM Patil Medical College and Hospital, BLDE University (Deemed to be University), Vijayapura, on 64 patients admitted for elective surgeries under general anesthesia lasting less than two hours. After approval by the Institutional Ethical Committee of BLDE University (approval number: IEC/09/2021), patients who were between the ages of 20-60 years, of both sexes, undergoing elective surgeries including upper limb debridement, upper limb fracture reduction and plating, open abdominal tubectomy, tympanoplasty, open appendicectomy, American Society of Anesthesiologist (ASA) grades I and II, and body mass index (BMI) < 30 kg/m^2^ were included in the study. Patients who refused to participate in the study, had restricted mouth opening, burns and swellings in the neck region, previous surgeries in the neck, and high aspiration risk with poor pulmonary compliance were excluded from the study. Patients were accepted for the trial after a thorough preoperative evaluation. The patients were randomly divided by a computer-generated random number table into two equal groups of 32 each: Group A (PLMA group) and Group B (BM group). Figure 1 shows the Consolidated Standard of Reporting Trials (CONSORT) diagram depicting how patients were enrolled in our study.

Sample size calculation was based on the study by Kumar et al. [8]. The required minimum sample size was 32 per group (i.e., a total sample size of 64, assuming equal group sizes) for achieving a power of 99% and a significance level of 2% (two-sided) for detecting an actual difference in means between two groups.

Patients were explained the procedure during the preoperative visit and written informed consent was taken.

Procedure

The patients were kept in fasting for eight hours for solids and two hours for clear liquids before surgery. All patients were given a tab of ranitidine 150 mg the night before surgery. They were shifted into the operating room, where baseline measurements were acquired using standard monitoring equipment like a pulse oximeter, sphygmomanometer cuff, electrocardiography (ECG) leads, and end-tidal carbon dioxide ( EtCO_2_) after device insertion. An intravenous (IV) line was secured with a 20G cannula and injection (inj). Ondansetron 0.15 mg/kg IV, Inj. glycopyrrolate 0.2 mg IV, and Inj. midazolam 0.01 mg/kg IV was used to premedicate the patient. Patients were pre-oxygenated for three minutes, with 100% oxygen, before induction of anesthesia. According to body weight, the proper LMA size was chosen. Following preoxygenation for three minutes, they were induced with Inj. propofol 2-3 mg/kg IV and Inj. fentanyl 2 mcg/kg IV, Inj. atracurium 0.5 mg/kg to facilitate the SAD insertion.

**Figure 2:**
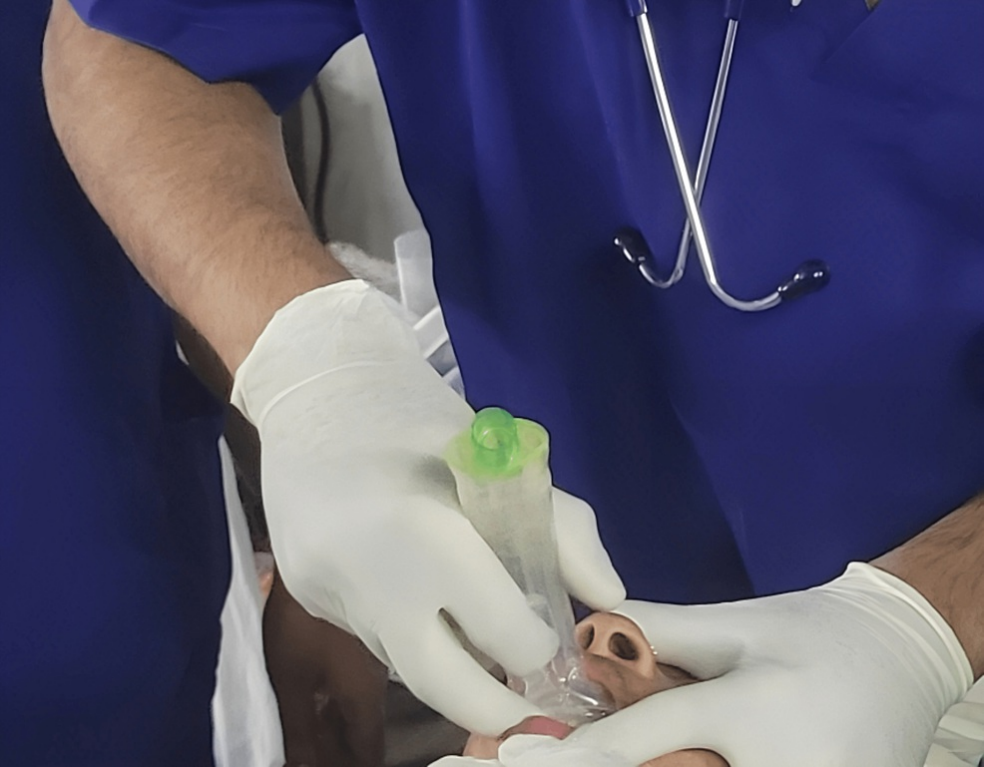
Insertion of Baska Mask

Before the start of the study, the principal investigator was trained in inserting the supraglottic airways BM and PLMA in adult patients. The device size was chosen according to the manufacturer's recommendations, as blinding was not possible during device insertion; data collection and analysis were done by the anesthesiologist blinded to the allotment. Both groups had a midline insertion procedure after adequate muscle relaxation, placing either of the devices in a neutral neck position. Patients allocated to Group A were secured an airway with PLMA, and patients in Group B were secured an airway with BM (Figure 2) (Proact Medical Systems, Frenchs Forest, New South Wales, Australia). The cuff of the PLMA was deflated fully, and the dorsal surface was lubricated with water-soluble jelly. Under adequate depth of anesthesia, the PLMA was held with the thumb and index finger and advanced around the palate pharyngeal curve until resistance was felt. The cuff was inflated with air according to the manufacturer’s recommendation. The ventral surface of the BM was lubricated with water-soluble jelly, BM was held between the thumb and index finger while insertion. The mask was pushed against the oropharyngeal curvature and hard palate till resistance was encountered. The device's angulation with the oropharyngeal curvature was altered using the tab for improved positioning. The tube of the airway device was connected to a closed circuit, and adequate ventilation was confirmed by bilateral symmetrical chest movements on manual ventilation, equal air entry on auscultation, and EtCO_2_ tracing of six square waveforms. Loss of less than 20% of set tidal volume on a ventilator, no gastric insufflations, and no audible leak at a peak airway pressure of 20 cmH2O during manual ventilation were also noted for correct placement of the device.

A maximum of three attempts were made to secure airways with the respective supraglottic device (either BM or PLMA) by a skilled anesthetist. Patients were intubated with an appropriate size of the endotracheal tube if the third attempt failed. Anesthesia was maintained with oxygen, nitrous oxide, and 0.6- 1% isoflurane, and an incremental dose of atracurium. EtCO_2_ of 35-45 mmHg was maintained.

An unblinded observer recorded the insertion time (the moment the device was taken into the operator's hands and successful ventilation was achieved is referred to as the time of insertion) and the number of attempts for successful insertion. Ease of insertion (Grade 1 - easy, Grade 2 - slightly difficult, Grade 3 - difficult, Grade 4 - impossible) was assessed by the resistance felt by the anesthetist while inserting the device. The OSP was measured in a neutral position five minutes post-insertion of the device. The fresh gas flow was kept at 6 L of oxygen, and adjustable pressure limiting (APL) valve was closed, i.e., at 40 cmH2O, at which equilibrium was reached, airway pressure was noted. The audible air leak was assessed by auscultation near the thyroid cartilage, and gastric insufflation was also checked during the leak pressure test by auscultation over the epigastric area. Intraoperatively, the patient was monitored for associated complications such as inadequate ventilation, which reflects poor chest expansion, absent or quiet breath sounds, absent or poor end-tidal CO_2_ trace, and fall in oxygen saturation; aspiration was identified by recognition of gastric contents in the oropharynx or airway, hypoxia, high airway pressures, and coarse crepitations on auscultation of the chest. At the end of the surgery, residual muscle relaxation was reversed by 0.05 mg/kg of neostigmine and 0.01 mg/kg of glycopyrrolate. When the patients were fully awake, the device was removed under continuous suction.

Post extubation, patients were monitored for laryngospasm, which presented as a fall in saturation and stridor, for which 100% oxygen was provided, IV corticosteroids were given, and if required, a sub-optimal dose of Inj. succinylcholine was given. In case of persistent laryngospasm, the patient was reintubated. Pharyngolaryngeal morbidity was evaluated by inspecting the device for blood stains and signs of visible trauma to the patient's lips, teeth, tongue, or oral tissue. These findings correlate with postoperative complications such as sore throat and hoarseness of voice.

Statistical analysis

Data were entered in the Microsoft Excel sheet (Microsoft Corporation, Redmond, Washington, United States), and statistical analysis was performed using IBM SPSS Statistics for Windows, Version 20.0 (Released 2011; IBM Corp., Armonk, New York, United States). Categorical variables like the number of attempts, gender distribution, and ASA grade was depicted by using counts and percentages. Demographic continuous variables such as age, BMI, duration of surgery, time of insertion, and ease of insertion were depicted using Mean ±SD, and comparison between these variables was done using the Mann-Whitney U test. The Chi-square test was used to determine the significant difference between qualitative data. To compare heart rate, mean arterial pressure, and oxygen saturation from baseline to end of the surgery, the Friedman test with post hoc test was performed. P value <0.05 was considered statistically significant.

## Results

The demographic profiles of the patients regarding age, BMI, ASA grade, and duration of surgery were comparable in both groups but statistically insignificant (Table 1).

**Table 1:** Comparison of Group A and Group B based on demographic data Group A: ProSeal laryngeal mask airway; Group B: Baska mask

Demographic variables	Group A	Group B (Mean + SD)	p-value
Age (years), mean±SD	34.19±11.544	30.84±10.568	0.183
BMI (kg/m^2^), mean±SD	22.2±1.65	21.59±1.47	0.125
Duration of surgery (minutes), mean±SD	59.38±28.65	53.13±25.708	0.334

BM was inserted in less time and with greater ease when compared to PLMA, which was statistically significant (Table 2). BM was also noted to provide a higher OSP than PLMA (Table 2), conferring the advantage of withstanding higher positive airway pressures.

**Table 2:** Comparison of Group A and Group B based on time taken for insertion, number of attempts, ease of insertion, and oropharyngeal seal pressure. *Statistically significant, p < 0.05 Group A: ProSeal laryngeal mask airway; Group B: Baska mask

	Group A	Group B	p-value
Time taken for insertion (seconds), mean±SD	28.59±1.682	24.00±1.136	0.001*
Number of attempts, N (%)			
1	22 (68%)	29 (90%)	0.0296*
2	10 (32%)	3 (10%)	
Ease of insertion, N (%)			
I	20 (62.5 %)	27 (84.4%)	0.007*
II	8 (25%)	5 (15.6%)	
III	4 (12.5%)	0 (0%)	
Oropharyngeal seal pressure (cmH2O), mean±SD	24.81±1.469	31.34±1.638	0.001*

A higher incidence of complications like trauma to the lip, blood staining, and sore throat was seen in patients where PLMA was used (Table 3). However, this was statistically insignificant.

**Table 3:** Incidence of complications in Group A and Group B Group A: ProSeal laryngeal mask airway; Group B: Baska mask

Trauma to lip	Group A	Group B	p-value
	N (%)	N (%)	
NO	27 (84.4)	31 (96.9)	0.08
YES	5 (15.6)	1 (3.1)	
Blood staining			
NO	27 (84.4)	30 (93.7)	0.2296
YES	5 (15.6)	2 (6.3)	
Sore throat			
NO	29 (90.6)	31 (96.9)	0.3017
YES	3 (9.4)	1 (3.1)	

## Discussion

Demographic profile regarding age, gender, BMI, ASA grade, and duration of surgery were comparable in both groups, which was not statistically significant (p>0.05). Both groups' hemodynamic parameters like mean arterial pressure, heart rate, and oxygen saturation were comparable and statistically insignificant (p >0.05).

The main observation of our study was the lesser time for insertion of the BM (24±1.136 seconds) compared to PLMA (28.59±1.682 seconds), which was statistically significant (p<0.001). Regarding ease of insertion, insertion was possible in 84.4% of patients in the BM group with no resistance (Grade I) compared to 62.5% of patients in the PLMA group. As a result, a higher success rate was observed in the first attempt with the BM than the PLMA (90% vs. 64%). The remaining patients required manipulation like jaw thrust and neck extension during the insertion of the second attempt. Four patients required a third attempt or tracheal intubation. A cuffless membrane of BM with an additional tap can be pulled to increase the curvature, aiding its insertion fast and efficiently into the oropharyngeal cavity [9]. Several studies over the years have favored our findings [10,11]. A study by Balwinderjit et al. found that, in terms of mean insertion time, the BM (14.25±3.82 seconds) took less time to insert than PLMA (22.01±2.64 seconds) [3]. Studies by van Zundert and Gatt [2] and Kumar et al. [8] also concluded similarly to ours.

SADs are classified into two types based on two critical distinctions. The first is whether or not an inflatable cuff is present [12,13]. Cuffless devices reduce the risk of cuff-related morbidity but may increase the risk of leaks and failure. First-generation devices are simple airway tubes with no particular design features aimed at reducing the risk of aspiration of gastric contents into the lungs. Second-generation SADs have additional modifications that help improve positive pressure ventilation (PPV) and lower the risk of aspiration into the lung [12].

Ramaiah et al. suggested a more widely accepted classification for SAD whose primary function is airway management under general anesthesia [13]. SAD were divided into categories according to the location of the device's cuff in the hypopharynx (and whether it is inflatable or anatomically preshaped), if it creates a seal, whether the effect of the seal is directed, and whether or not sealing of esophagus takes place: (i) Inflating mechanism with one or more cuffs, (ii) Preshaped devices that fit into the position, and (iii) Automatic or self-energizing devices, with the transmission of airway pressure to the inside of a flexible sealing element [14,15].

Due to their adaptability and simplicity of use, first-generation SADs quickly replaced endotracheal intubation and face masks in more than 40% of patients undergoing general anesthesia. By incorporating unique design enhancements such as being able to withstand higher airway pressure, composition by disposable materials, bite block, capacity to act as tracheal tube conduits, and decreased risk of aspiration of gastric contents to lungs, second-generation devices have increased their efficacy and utility [12,13].

The PLMA is made of medical-grade silicone and offers several benefits due to the following new features which include ventral cuff, which seals the peri glottic tissues and improves the seal, gastric port for suctioning of gastric contents, integrated bite blocks, an anterior distal tube fitted with a locating strap which helps in avoiding the finger from slipping off the tube, more oversized ventral cuff, accessory vent that prevents secretions from collecting, configuration with two tubes and an airway tube reinforced with wire. The PLMA can be reused; its product life is nearly 40 sterilizations [16,17]. The third-generation BM is the newest member of the SAD family and can endure high airway pressures while reducing aspiration and laryngeal trauma. The BM replaces the orogastric tube with a sump and two drains. The BM combines elements from (i) the PLMA, which provides more significant oropharyngeal seal pressure, ports for gastric suctioning and bite block, (ii) the LMA® Supreme™ (Teleflex Incorporated), which includes a drain for gastric contents, (3) the i-gel® (Intersurgical Ltd, Wokingham, United Kingdom), and (4) the Streamlined Liner of the Pharynx Airway (SLIPA™) (Teleflex Incorporated), which has a sump reservoir. The bite block of the BM covers the entire curvature of the airway tube. It has a similar oval shape to the mouth, which prevents it from spinning in the pharynx [9]. More than 90% of patients who cannot receive tracheal intubation or mask ventilation can receive successful rescue ventilation using SADs [18].

The SAD with high OSP is required to provide positive pressure ventilation and help in preventing aspiration. Our study showed significant (p < 0.001) results with higher OSP in the BM (31.34±1.638 cmH2O) compared to PLMA (24.81±1.469 cm H2O) (Table 1). A study by Al-Rawahi et al. recorded similar results, which are 29.98±8.51 cmH2O for the BM and 24.05±6.19 cmH2O for PLMA [19]. Another study by Agrawal et al. also was in agreement with our research with mean seal pressures of 37.6±2.43 cmH2O for BM and 30.82±3.96 cmH2O for PLMA [7]. BM due to its recoiling cuff during each positive pressure ventilation, inflates and deflates, thereby increasing the seal pressure during positive pressure ventilation.

Complications associated with insertion trauma to the lip, blood staining, and sore throat were more in the PLMA group (15.6%, 15.6%, and 9.4%, respectively) compared to patients inserted with the BM group (6.3%, 3.1%, and 3.1%, respectively) (Table 2). These results were, however, statistically insignificant (p-value >0.05). A study by Agrawal et al., however, showed no adverse incidence-like aspiration of gastric contents, staining of blood on the removal of the SAD, attributing it to the gentle insertion by skilled anesthetists, use of jelly for lubricating the surface, and that the PLMA cuff pressure was kept below 60 cmH2O throughout the surgery [7]. On the other hand, a study by Zundert et al. revealed that 2% of patients had lip damage and 8% had a blood stain on the BM [2]. A study by Al-Rawahi et al. also had similar observations to our research [19]. In our study, we did not observed any complications like dysphagia, hoarseness of voice, or gastric contents aspiration after removing the LMA.

Limitations

This study had some limitations. The study was done in a single center with limited patients. ASA grade III and IV patients and patients with difficult airways were excluded. Second, the patients involved in this study were single-centered and there was heterogeneity in the surgery performed. As a result, more research may be required to complete the validation analysis based on the findings of this investigation.

## Conclusions

BM placement has significantly less insertion time with ease and a higher first-attempt success rate compared to PLMA. It also provides better OSP and protects the airway from aspiration with fewer complications. Hence, our study emphasizes the advantages of using the BM over PLMA, encouraging its use in clinical practice.

## References

[1] White LD, Thang C, Hodsdon A, Melhuish TM, Barron FA, Godsall MG, Vlok R (2020). Comparison of supraglottic airway devices with endotracheal intubation in low-risk patients for cesarean delivery: systematic review and meta-analysis. Anesth Analg.

[2] Van Zundert T, Gatt S (2021). The Baska Mask®-a new concept in self-sealing membrane cuff extragalactic airway devices, using a sump and two gastric drains. A critical evaluation. J Obstet Anaesth Crit Care.

[3] Almeida G, Costa AC, Machado HS (2016). Supraglottic airway devices: a review in a new era of airway management. J Anesth Clin Res.

[4] Hussain D, Kundal R, Kumar A, Sabharwal N (2022). An analysis of the comparative efficacy between a third-generation and a second-generation supraglottic airway device in patients undergoing laparoscopic cholecystectomy. Cureus.

[5] Shiveshi P, Anandaswamy TC (2022). Comparison of Proseal L.M.A. with i-Gel in children under controlled ventilation: a prospective randomized clinical study Braz. J Anesthesiol.

[6] Reddy P, Shivkumara KC, Madhu M, Osheen M (2022). Baska mask, i-Gel supraglottic airway device and LMA Proseal in spontaneously breathing, anesthetized children during elective surgeries: ease of insertion of the three devices. Eur J Mol Clin Med.

[7] Agrawal N, Singh A, Gupta A (2022). Comparative study of Baska mask with proseal LMA in adult patients undergoing elective surgery under general anesthesia with controlled ventilation. J Anaesthesiol Clin Pharmacol.

[8] Kumar EJ, Anand GV, Shaji RA (2019). A comparative study of Baska mask vs proseal LMA in elective sterilization surgeries. Int Arch Integr Med.

[9] Chauhan G, Nayar P, Seth A, Gupta K, Panwar M, Agrawal N (2013). Comparison of clinical performance of the I-gel with LMA proseal. J Anaesthesiol Clin Pharmacol.

[10] Kachakayala RK, Bhatia P, Singh S, Dwivedi D (2020). A comparative study of supraglottic airway devices Baska mask and ProSeal-laryngeal mask airway in short gynecological procedures. J Int Med Res.

[11] Garpagalakshmi S (2019). A Comparative Study of Baska Mask and Proseal Laryngeal Mask for General Anaesthesia With Intermittent Positive Pressure Ventilation (Doctoral Dissertation). http://repository-tnmgrmu.ac.in/11040/1/201000219garpagalakshmi.pdf.

[12] Ramachandran SK, Kumar AM (2014). Supraglottic airway devices. Respir Care.

[13] Ramaiah R, Das D, Bhananker SM, Joffe AM (2014). Extraglottic airway devices: a review. Int J Crit Illn Inj Sci.

[14] Parameswari A, Dhanasekaran R, Mehta GD (2019). A prospective randomized comparative study between Baska mask, Proseal L.M.A. and I Gel during positive pressure ventilation in laparoscopic cholecystectomy. Anestezi Dergisi.

[15] Bein B, Scholz J (2005). Supraglottic airway devices. Best Pract Res Clin Anaesthesiol.

[16] Cook TM, Lee G, Nolan JP (2005). The ProSeal laryngeal mask airway: a review of the literature. Can J Anaesth.

[17] Brimacombe J, Keller C (2000). The ProSeal laryngeal mask airway: a randomized, crossover study with the standard laryngeal mask airway in paralyzed, anesthetized patients. Anesthesiology.

[18] Alexiev V, Salim A, Kevin LG, Laffey JG (2012). An observational study of the Baska® mask: a novel supraglottic airway. Anaesthesia.

[19] Al-Rawahi SA, Aziz H, Malik AM, Khan RM, Kaul N (2013). A comparative analysis of the Baska® Mask vs. Proseal laryngeal mask for general anesthesia with I.P.P.V. Anaesth Pain Intensive Care.

